# *ACTN3* XX Genotype Negatively Affects Running Performance and Increases Muscle Injury Incidence in *LaLiga* Football Players

**DOI:** 10.3390/genes15030386

**Published:** 2024-03-21

**Authors:** Juan Del Coso, Gil Rodas, Aitor Soler-Aguinaga, Roberto López-Del Campo, Ricardo Resta, Joaquín González-Rodenas, Jordi Ferrandis, Víctor Moreno-Pérez

**Affiliations:** 1 Sport Sciences Research Centre, Rey Juan Carlos University, 28943 Fuenlabrada, Spain; joaquin.gonzalez@urjc.es (J.G.-R.); jordi.ferrandis@urjc.es (J.F.); 2 Medical Department & Barça Innovation Hub, Fútbol Club Barcelona, 08038 Barcelona, Spain; gil.rodas@fcbarcelona.cat; 3 Strength and Conditioning Department, Elche Club de Fútbol, 03208 Elche, Spain; aitorjsoler@gmail.com; 4 Department of Competitions, La Liga, 28043 Madrid, Spain; rlopez@laliga.es (R.L.-D.C.); rresta@laliga.es (R.R.); 5 Faculty of Physical Education and Sports Sciences, Catholic University of Valencia, “San Vicente Mártir”, 46001 Valencia, Spain; 6 Department of Sport Sciences, Sports Research Centre, Miguel Hernandez University of Elche, 03202 Elche, Spain; vmoreno@umh.es; 7 Department of Pathology and Surgery, Translational Research Centre of Physiotherapy, Faculty of Medicine, Miguel Hernandez University, 03202 Elche, Spain

**Keywords:** *ACTN3* gene, rs1815739, soccer, genetic variation, team sports, physical performance

## Abstract

The aim of this study was to investigate the association of the *ACTN3* rs1815739 polymorphism with match running performance and injury incidence in top-level professional football players. A total of 315 top-level professional football players from the first division of Spanish football (i.e., *LaLiga*) participated in this prospective and descriptive study. The *ACTN3* rs1815739 genotype was identified for each player using genomic DNA samples. During *LaLiga* 2021–2022, players’ performance was obtained through a validated camera system in all official matches. Additionally, the incidence of non-contact injuries was obtained by each team’s medical staff according to the International Olympic Committee (IOC) statement. From the study sample, 116 (36.8%) players had the RR genotype, 156 (49.5%) had the RX genotype, and 43 (13.7%) had the XX genotype. The anthropometric characteristics of the players were similar across genotypes. However, the total running distance (*p* = 0.046), the distance at 21.0–23.9 km/h (*p* = 0.042), and the number of sprints (*p* = 0.042) were associated with the *ACTN3* genotype. In all these variables, XX players had lower match performance values than RR players. Additionally, total and match injury incidences were higher in XX players than in RR players (*p* = 0.026 and 0.009, respectively). The rate of muscle injuries was also higher in XX players (*p* = 0.016). *LaLiga* football players with the *ACTN3* XX genotype had lower match running performance and a higher incidence of non-contact injuries over the season.

## 1. Introduction

α-Actinin-3 is a structural protein within the skeletal muscle that anchors actin filaments to the Z-disc to regulate muscle length and tension during contraction [1]. α-actinin-3 is expressed only in fast type two muscle fibers, suggesting a specific function for powerful and fast muscle contractions [2]. α-actinin-3 is encoded by the *ACTN3* gene, which contains a single nucleotide polymorphism (rs1815739, also known as the p.R577X polymorphism) that leads to the replacement of an arginine (R) with a premature stop codon (X) at amino acid 577. The result of this polymorphism is that individuals with two copies of the 577X allele (XX genotype) are deficient in α-actinin-3 as they do not express the protein. In contrast, homozygous individuals for the 577R allele (RR genotype) or heterozygote individuals (RX genotype) express α-actinin-3, although there is dose-dependent expression of α-actinin-3 in R allele carriers (expression of RR > RX) [3]. 

The deficiency of α-actinin-3 does not entail any disease or clinical condition because there is another α-actinin isoform, the α-actinin-2, that completes a similar role within the muscle. In fact, about 18% of the world’s population possesses the XX genotype [4]. However, previous investigations have demonstrated that the deficiency of α-actinin-3 due to the *ACTN3* XX genotype may induce some potentially negative phenotypes in humans, such as lower muscle strength [5], reduced muscle volume [6], higher levels of muscle damage induced by exercise [7] and diminished bone mineral density [8].

*ACTN3* R577X genetic variation is one of the most investigated polymorphisms in athletes. Overall, several investigations have found that XX athletes are underrepresented in elite power-oriented sports [1]. Specifically, these studies revealed that there was a lower frequency of the 577XX genotype in elite sprint/power athletes compared to the non-athletes [1,4,9]. On the contrary, these same studies reported a higher frequency of the 577RR genotype in elite sprint/power athletes compared to the general population. For this reason, the *ACTN3* is often called the “speed gene” as sprint/power-oriented athletes in both genders and across different sports and ethnic backgrounds have lower frequencies of the *ACTN3* XX genotype [1]. Interestingly, the phenotype(s) associated with the underrepresentation of XX athletes in power-based sports disciplines has(ve) not been clearly identified. However, according to the authors’ opinions, the lower physical performance reported in several sprint activities [10], the higher values of exercise-induced muscle damage after stressful exercise activities [11] and the higher injury incidence in XX athletes [12] in comparison to RR athletes are likely contributors for the underrepresentation of XX athletes in power-based sports disciplines.

Among team sports, football seems an ideal sport to investigate the effect of the *ACTN3* R577X polymorphism, as football is a highly intense sport characterized by the repetition of near-to-maximum exercise actions, such as accelerations/decelerations, short sprints and changes in direction [13]. A current meta-analysis including 17 studies about the association of the R577X polymorphism with the athlete status in football concluded that the frequency of XX players was lower than in non-athlete populations [14]. Additionally, it has been found that XX players had a higher predisposition to muscle injuries than RR players [12,15,16] which may also contribute to the limitation of XX individuals to become professional football players. However, these investigations were carried out with small samples, with professional football players of secondary football leagues and/or without the measurement of any football-specific performance phenotype. Hence, the true association of the *ACTN3* genotype with football players’ performance and injury incidence in top-tier football leagues has not been properly investigated.

Therefore, the aim of this study was to investigate the association of the *ACTN3* R577X polymorphism with match running performance and injury incidence of top-level professional male football players of *LaLiga*. We selected the football players competing in *LaLiga* as this competition has been deemed as the national football competition with the highest indices of international prestige and competitive quality [17]. We hypothesized that XX football players would be underrepresented in *LaLiga*, particularly in best-ranked teams, would have lower match running performance and would have higher muscle injury rates than RR football players. 

## 2. Materials and Methods

### 2.1. Participants

A total of 315 professional football players from 12 teams competing in the first division of football in Spain (i.e., *LaLiga Santander*) participated in this prospective and descriptive investigation. Among these, 273 were players of Caucasian descent, 26 were players of African descent, and 1 was a player of Asian descent. In the sample, there were 34 goalkeepers, 56 centre backs, 54 full backs, 80 midfielders, 41 wingers and 50 strikers. In the study sample, 11 players had won a FIFA World Cup and 14 players had won a UEFA European Championship obtained with their national teams; 13 players had won at least one UEFA Champion’s League and 11 had won at least one UEFA Europa League with their clubs. Before the start of this investigation, written informed consent was obtained from all of the players. The procedures were approved by an institutional Ethics Review Committee (DPC.VMP.01.20) and are in accordance with the consensus statement of the International Federation of Sports Medicine for genetic information [18]. All players received an individual report with information about their *ACTN3* genotype as compensation for their participation in the study.

### 2.2. Measures

#### 2.2.1. Genetic Testing

Genetic data were collected from April to September 2021. During a training session in the team’s facilities, one member of the research group explained the objective of the investigation and answered all the questions that players had about the use of the samples. After this, all participants signed the written informed consent and completed an ad hoc questionnaire with personal and training information. Afterwards, anthropometric variables (i.e., body mass and height; ±50 g scale; Radwag, Poland) were measured and a sample of genomic DNA was obtained by buccal smear with a cotton swab. The DNA samples were identified with an alphanumeric code to avoid the identification of the participants. At a later date (no more than 30 days after sample collection), the genomic DNA of each sample was isolated following standardized protocols [19] and genotyping was performed in a certified genetics laboratory. To avoid contamination, recommendations for molecular genetics laboratories were followed, including physically isolated work area laboratories for each process (sample manipulation and extraction). In addition, reference samples (internal controls, blank samples, and negative controls) and contamination monitoring in all steps were included. Specifically, genomic DNA was isolated using an organic-based DNA extraction method adapted to Amicon^®^ Ultra-0.5 mL columns (Sigma-Aldrich, Madrid, Spain), including an elution of the sample in 50 µL of interval control to obtain a solution with a DNA concentration of ~30 ng/mL. Positive controls for all genotypes were used from the Mexican branch of CANDELA Consortium [20]. Genotyping of *ACTN3* rs1815739 polymorphism (c.1858C>T; p.R577X) was conducted using a TaqMan SNP Genotyping Assay (Assay ID: C___590093_1_; Applied Biosystems) and the reaction was performed in an Applied Biosystems 7500 Fast Real-Time PCR System (Applied Biosystems, Foster City, CA, USA). The results were analyzed using 7500 Software v2.0.5 (Applied Biosystems). DNA analyses that did not reported a clear *ACTN3* genotype were repeated. From the total, 60 samples were randomly selected, and they were genotyped a second time. We confirmed that the genotyping results perfectly agreed between duplicates which reinforces the accuracy of the analysis. The laboratory provided an identification of the genotype for each alphanumeric code/sample and the researchers were then responsible for linking the information from the laboratory with the players’ data to avoid the identification of players’ identities during the genotyping process.

#### 2.2.2. Running Performance during Official Matches

To develop an analysis of the effect of the *ACTN3* genotype on football performance with the highest standards of ecological validity, running performance was obtained from all players in the matches played in the 2021–2022 season (from 13 August 2021, to 22 May 2022) using the multicamera Mediacoach^®^ system (Spain). Mediacoach is a valid tracking system that evaluates the position of each player during match play with a sampling frequency of 25 Hz. Mediacoach is composed of two multi-camera units placed at either side of the midfield line with a resolution of 1920 × 1080 pixels, which are synchronized to provide a stitched panoramic picture. The panoramic picture is then employed to create the stereoscopic view that allows for triangulating all the players on the field to assess their position and calculate instantaneous running speed during the match. Validation of Mediacoach to assess player’s running speed and running distance during official matches has been certified through a high agreement with the data obtained with Global Positioning System units [21] and with data obtained from a reference camera system [22]. After each match, Mediacoach automatically produced a report for each player that has participated in the match with information about running distance in total and at different speeds (distance at 2.0–6.9 km/h, at 7.0–13.9 km/h, at 14.0–20.9 km/h, at 21.0–23.9 km/h, and at ≥24 km/h). The report also contained information about the number of sprints at ≥24 km/h, the duration and distance of the sprints and the peak velocity reached in each sprint. The information used for this investigation contains the accumulated value for each player as a result of their participation in all *LaLiga* matches during the whole season. As not all players had the same match exposure during the season, all match running performance variables were normalized by match exposure (in min). For this analysis, players with less than 90 min of match exposure during the whole season were removed, as their running data were likely not representative of their match performance. Additionally, goalkeepers were removed from the analysis of match running performance variables as their running patterns during the match were different and not comparable to the remaining field positions.

#### 2.2.3. Injury Data Collection

All non-contact injuries that occurred during the 2021–2022 season were recorded by the medical staff of each team participating in the investigation. Contact injuries, defined as those injuries produced by the collision with another player or with an object were rejected from the analysis. Non-contact injuries were diagnosed, and classified following the 2020 consensus statement for methods of recording and reporting epidemiological data on injury and illness in sport developed by the International Olympic Committee [23]. This included classification of each injury according to its mode of onset (acute or gradual), type (muscle, tendon, ligament, bone, cartilage, nerve or other), body location, exposure (match vs. training) and recurrence. Injury severity was categorized as slight if return to play was within 1–3 days after injury, mild (return to play was within 4–7 days), moderate (return to play was within 8–28 days), or severe (return to play was longer than 28 days). Injury incidence (overall and match and training injury incidences), as number of injuries per 1000 h of football exposure, was individually calculated by the research team for each football player. For this calculation, we used the number of non-contact injuries reported by the medical staff of the teams and football exposure times (match and training exposures) reported by the strength and conditioning staff of the teams.

### 2.3. Statistical Analyses

We determined whether the *ACTN3* genotype distribution in the sample met the Hardy–Weinberg Equilibrium (HWE) using a χ^2^ test. A χ^2^ test was also used to verify if the genotype frequency in our cohort of football players was different from the 1000 Genomes database of ethnically matched controls [24]. Descriptive statistics were calculated for each genotype as frequencies for categorical variables and as mean ± standard deviation for continuous variables. Differences among genotypes for categorical variables (e.g., race or field position) were determined with χ^2^ tests. In the case of a significant χ^2^ test, standardized residuals were calculated to identify genotypes with observed distribution different from the expected distribution. For continuous variables (e.g., running performance variables or anthropometric variables), normality was initially checked with the Kolmogorov–Smirnov test, and differences among genotypes were calculated with a one-way analysis of variance (ANOVA) to determine the main effect of the *ACTN3* genotype (all variables had a normal distribution). In the case of a main effect of the genotype due to a significant F test, LSD post hoc tests were applied to identify pairwise differences (RR vs. RX; RR vs. XX; RX vs. XX). Additionally, the effect of a dominant model for the *ACTN3* genotype (RR vs. RX + XX) and a recessive model (RR + RX vs. XX) was calculated for each running performance variable using unpaired Student’s *t*-tests. The level of significance for all statistical analyses was set at *p* < 0.050. All statistical analyses were performed with statistical software (SPSS Statistics 27, IBM, Armonk, NY, USA). 

## 3. Results

### 3.1. Sociodemographic Variables

The rate of genotyping success was 100%, with the following genotype distribution (which were in HWE, *p* = 0.709): RR, 36.8%; RX, 49.5%; and XX, 13.7% (Table 1). The genotype frequencies in our sample of professional football players were comparable to the 1000 Genomes database for the Caucasian population (*p* = 0.122). However, our sample of football players of African descent differed from the general data for the African population reported in the 1000 Genomes database (*p* < 0.001), with a higher proportion of players with the RR genotype and a lower proportion of players with the RX genotype (both *p* < 0.005). There were no differences in age, body mass, body height or body mass index across genotypes. However, the distribution of genotypes in players of African descent differed from that of players of Caucasian descent, as the African descent subsample had an unexpectedly higher proportion of players with the RR genotype (73.1% in Africans and 33.7% in Caucasians; *p* < 0.001) and a lower proportion of players with the RX genotype (23.1% in Africans and 51.7% in Caucasians; *p* = 0.002) in comparison to their Caucasian counterparts.

### 3.2. Field Position

**Figure 1:**
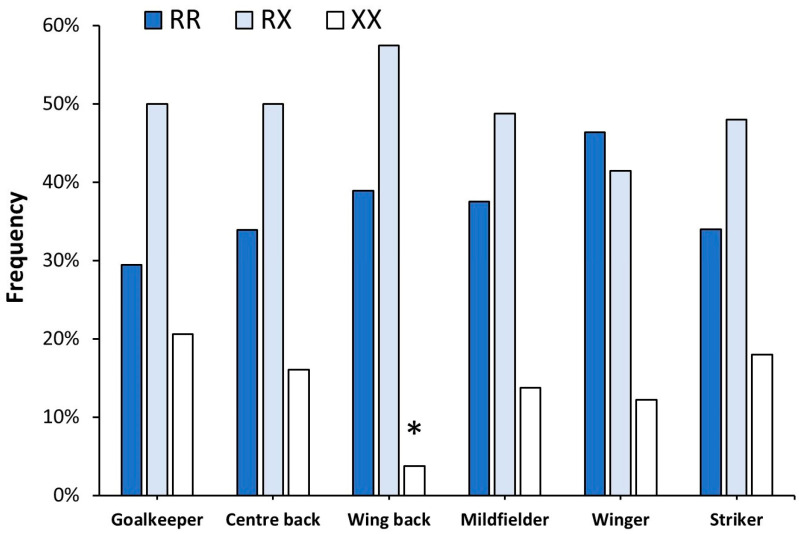
Distribution of *ACTN3* genotypes in 315 professional football players competing in *LaLiga* depending on their field position. The * indicates that the observed distribution was lower than the expected distribution at *p* < 0.050, identified with the χ^2^ test.

Figure 1 depicts the *ACTN3* R577X genotype distribution depending on field position. Overall, there were no statistically significant differences in the distribution of genotypes among the different field positions (*p* = 0.514). However, the proportion of XX wing back players was lower than expected (*p* = 0.040).

### 3.3. Running Performance during Official Matches

**Figure 2:**
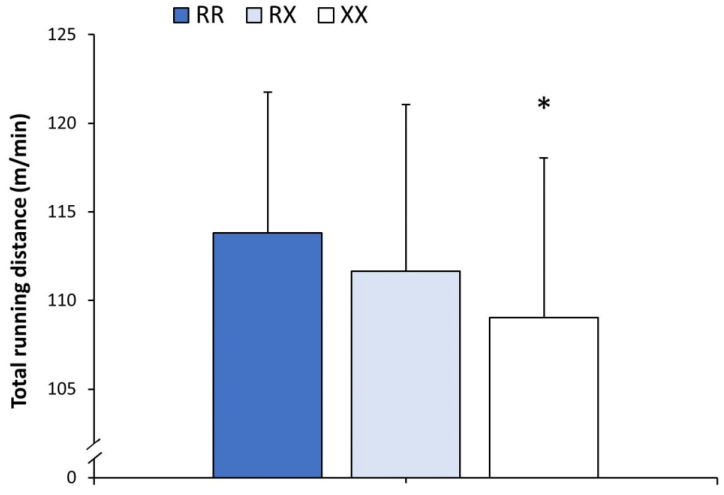
Match running distance in official matches of *LaLiga* 2021–2022 depending on players’ *ACTN3* genotype. Total running distance was obtained in a subsample of 259 football players after removing players with less than 90 min of match exposure during the whole season and goalkeepers. The * depicts a difference between players with XX genotype vs. players with RR genotype at *p* < 0.050, identified with one-way ANOVA.

**Figure 3:**
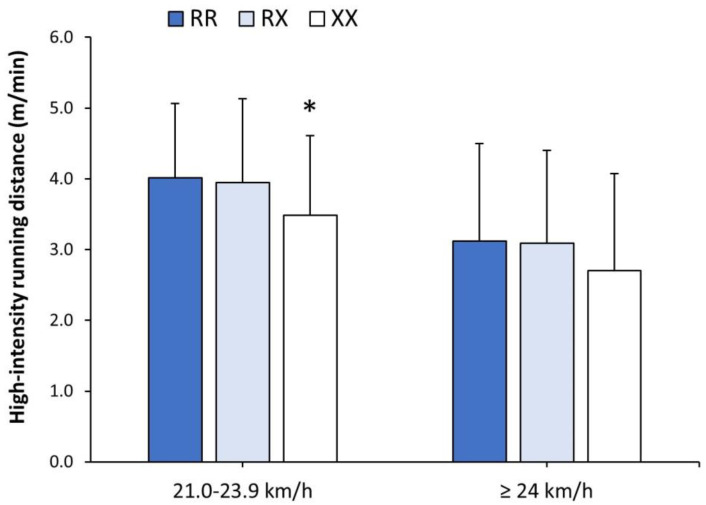
Match running distance at high intensity in official matches of *LaLiga* 2021–2022 depending on players’ *ACTN3* genotype. High intensity running distance was obtained in a subsample of 259 football players after removing players with less than 90 min of match exposure during the whole season and goalkeepers. The * depicts a difference between players with XX genotype vs. players with RR genotype at *p* < 0.050, identified with one-way ANOVA.

There was a main effect of the genotype on the total distance run during the season (Figure 2). *ACTN3* XX players ran 4.2% less distance than their RR counterparts (*p* = 0.023), with no differences between RX and RR players. Additionally, the dominant model (RR vs. RX + XX, 113.8 ± 7.9 vs. 111.0 ± 10.8 m/min, respectively; *p* = 0.032) and the recessive model (RR + RX vs. XX, 112.7 ± 8.0 vs. 109.0 ± 9.0 m/min, respectively; *p* = 0.047) for the *ACTN3* genotype presented differences in total running distance. There was no main effect of the genotype on the running distance covered at 2.0–6.9 km/h, 7.0–13.9 km/h and 14.0–20.9 km/h (Table 1), but the effect of the *ACTN3* genotype was observed on the distance run at a speed of 21.0–23.9 km/h. Specifically, XX players covered 13.1% less distance at 21.0–23.9 km/h than RR players (*p* = 0.021; Figure 3). XX players also ran 13.4% less distance at ≥24.0 km/h than RR players, but the difference did not reach statistical significance (*p* = 0.101). Only the recessive model reached statistical significance for the distance covered at 21.0–23.9 km/h (RR + RX vs. XX, 4.0 ± 1.1 vs. 3.5 ± 1.1 m/min, respectively; *p* = 0.026). 

There was a main effect of the genotype in the number of sprints performed during official matches (Table 1). Football players with the XX genotype performed 14.3% less sprints than players with the RR genotype (*p* = 0.047) and 14.1% less sprints than players with the RX genotype (*p* = 0.050). As a result, the recessive model reached statistical significance for the number of sprints (RR + RX vs. XX, 0.23 ± 0.08 vs. 0.20 ± 0.08 sprints/min, respectively *p* = 0.042). Additionally, there was a main effect of genotype on sprint time as the XX players performed sprints of shorter duration than RR players (*p* = 0.031). However, there was no main effect of the genotype on peak running velocity or mean sprint distance. The match time played during the 2021–2022 season and the number of matches played as a starter or as a substitute was unaffected by the *ACTN3* genotype (Table 1). 

### 3.4. Team Performance

The distribution of the *ACTN3* genotype was similar in teams ranked in the top 10 vs. the bottom 10 at the end of the season (*p* = 0.101). However, all teams ranked in the top 10 had squads with a proportion of XX players lower than 10% (8.2%, on average for top 10 teams). Additionally, the only three teams that had a proportion of XX players above 20% were ranked in the bottom 10 at the end of the season (16.6% for all the teams ranked in the bottom 10). 

### 3.5. Injury Incidence

**Figure 4:**
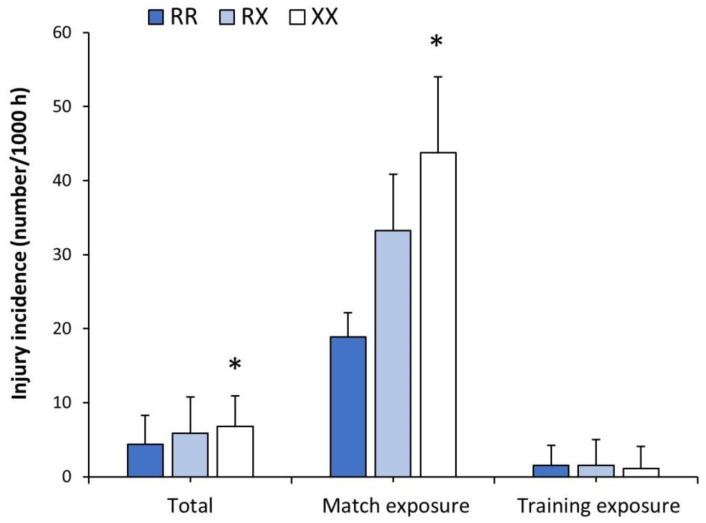
Injury incidence in 315 professional football players competing in *LaLiga* depending on their *ACTN3* genotype. Match injury incidence was obtained in a subsample of 259 football players after removing players with less than 90 min of match exposure during the whole season and goalkeepers. The * depicts a difference between players with XX genotype vs. players with RR genotype at *p* < 0.050, identified with one-way ANOVA.

There was an effect of the *ACTN3* genotype on the total injury incidence and match injury incidence, with no effect of the genotype on training injury incidence (Table 2). Specifically, total and match injury incidences were higher in XX players than in RR players (*p* = 0.026 and 0.009, respectively; Figure 4). The dominant model presented differences for total injury incidence (RR vs. RX + XX, 3.4 ± 3.9 vs. 5.0 ± 5.5 injury per 1000 h, respectively; *p* = 0.044) and match injury incidence (RR vs. RX + XX, 18.9 ± 3.3 vs. 35.6 ± 8.2 injury per 1000 h, respectively; *p* = 0.021) with no differences for the recessive model. The incidences of both muscle injury and bone injury were also affected by the *ACTN3* genotype. In both cases, XX players presented higher rates of injury for muscle (*p* = 0.016) and bone (*p* = 0.033) injuries than RR players, without differences among genotypes in the other types of injuries. The number of days that players needed to return to play after each type of injury was unaffected by the *ACTN3* genotype (Table 2). 

## 4. Discussion

Several previous investigations, summarized in the meta-analysis by McAuley et al., [14] found that the frequency of football players with the XX genotype in the *ACTN3* gene in professional teams is lower than the frequency of individuals with the XX genotype in the general population. This underrepresentation of *ACTN3* XX professional football players suggests the XX genotype may be somewhat deleterious to reach the status of a professional football player. However, the case–control studies summarized by McAuley et al. [14] do not provide enough information to determine what specific phenotype associated with the XX genotype produces a lower presence of XX players in professional football teams. To date, no previous investigation has measured phenotypes associated with reaching the status of elite/professional football players to ascertain why the XX genotype is underrepresented in professional football. For this reason, the aim of this study was to investigate the association of the *ACTN3* rs1815739 polymorphism (and its three genotypes) with match-running performance and injury incidence of top-level professional football players. This study was designed to solve some of the drawbacks of previous investigations on this topic as we recruited football players from a truly selective national football league, and we assessed several football-specific performance phenotypes as high-intensity running during official football matches and injury incidence. The current study is novel because we have found that *ACTN3* XX football players ran less distance than their RR counterparts during official matches over one season of *LaLiga*. The lower running performance of XX football players was particularly present at higher speeds, which showed that XX players performed a smaller number of sprints with shorter sprint durations than RR players. These data indicate that XX players were involved in a lower number of sprint actions during official matches, and they were incapable of maintaining these sprint efforts for a similar time to RR players. In addition, the frequency of injuries in football players with the XX genotype was higher than that of RR players, especially for the injuries that occurred during matches and those located in muscle tissue. Interestingly, most of the phenotypes measured in this investigation presented an RR > RX > XX arrangement (and the contrary for injury incidence) which coincides with the progressive expression of α-actinin-3 of the different *ACTN3* genotypes [3]. In light of these data, we hypothesize that the *ACTN3* rs1815739 polymorphism is somewhat associated with football performance as top-level XX players may exhibit lower match running performance and injury incidence than RR their counterparts.

### 4.1. ACTN3 and Running Performance

Football is an intermittent team sport that demands high-intensity actions such as sudden sprints, rapid changes of direction and, in many situations, kicking the ball. These actions are habitually performed near the ball and require high values of speed, strength, power, and agility [25]. During a competitive match, football players run around ~110 m per minute and perform a sprint every ~90 s [26]. Most of these high-intensity runs are shorter than 20 m, while the maximum sprinting velocity of professional football players is normally between 31 and 32 km/h [27]. The current analysis reflects that all football players fulfilled these high-performance running patterns irrespective of their *ACTN3* genotype, which confirms that all football players in our sample were exceptional athletes. However, subtle differences were found to be associated with the *ACTN3* rs1815739 polymorphism, as XX players had lower values in several match running variables, particularly regarding their capacity to run and sprint. It is interesting to note that football squads with the best ranking at the end of the season (i.e., classified among the top-ten teams in *LaLiga*) presented only 8.2% of XX football players, well below the ~18% of XX individuals in general Caucasian populations [24]. Taken together, this information suggests that top-level football players with the *ACTN3* XX genotype may have a slightly lower physical match performance than their RR counterparts which is potentially linked with the underrepresentation of XX players in professional leagues of football [14]. In the authors’ opinion, this does not entail that XX players cannot reach the status of elite professional players, as they are in fact included in our sample of professional football players. Furthermore, it seems that this subtle lower running performance of XX players did not influence the manager’s/coach’s decisions to select which players are on the pitch, as the number of matches played as a starter/substitute and the overall match time during the season was similar across genotypes. In summary, if these causal links between the *ACTN3* XX genotype and lower match running performance are confirmed, it may suggest that professional XX football players compensate for their slightly lower capacity to run/sprint with other key football capacities such as higher technical and tactical skills, which constitutes an excellent hypothesis for further investigations. An alternative hypothesis is that the XX genotype would provide elite players with superior aerobic capacity to compensate for their lower anaerobic capacity, considering some evidence of XX genotype overrepresentation in elite endurance athletes [4].

### 4.2. ACTN3 and Injury Incidence

Football offers extensive health benefits for cardiovascular, metabolic and musculoskeletal fitness [28]. However, the intense nature of football produces that this team sport is associated with high injury rates, especially at the professional level. The incidence of injury in professional male football players is ~8.1 injuries/1000 h of exposure, with a several-fold higher injury incidence during matches compared to training exposure (36.0 vs. 3.7 injuries/1000 h of exposure, respectively) [29]. The previous literature has suggested the existence of several non-modifiable risk factors for injury in football and other high-intensity sports such as athlete’s age, the existence of a previous injury of the same type and in the same body location, and the architecture of the muscle [30], among others. Fortunately, there are also modifiable factors for the risk of injury like muscle flexibility and joint range of motion [31], chronic and acute fatigue [32] or low muscle strength [33]. The current investigation contributes to the literature on this topic as shows that the *ACTN3* genotype may also be a contributing factor to football-specific injury. Several recent studies conducted on professional football players [15,16] have found that XX football players were more prone to injury. The novelty of the current investigation is that we have been able to identify that the higher injury incidence of XX football players may be associated with their higher susceptibility to muscle and bone injuries during matches with no difference in other types of injuries or during training exposure. The potential deleterious effect of the XX genotype for increasing injury rates only during match exposure is probably linked to the particularities of current professional football. The complex fixture over the season with several national and international competitions suggests that most of the training sessions in the week have a recovery objective (and, therefore, a very limited external load) as professional teams habitually compete in two matches per week. This likely affected the fact that the differences in injury rates associated with the *ACTN3* genotype were only present during match exposure. Regarding the injury type, XX players only had a higher incidence of muscle and bone injuries with respect to their RR counterparts. This pattern of increased muscle injury incidence in XX football players may be potentially linked with the effect of *ACTN3* rs1815739 polymorphism in humans, as the XX genotype produces the lack of a structural protein within the muscle (i.e., α-actinin-3 deficiency) in fast-type muscle fibers, which has been deemed as essential to resist high levels of muscle stress [9]. Additionally, there is evidence suggesting that XX humans also have decreased bone mineral density [8], which may have contributed to the higher bone injury incidence in XX players. However, the explanation for the higher bone injury rate in XX players requires further support as the lower bone mineral density in XX individuals has only been established in postmenopausal women [8]. Last, in the current study, the return-to-play process was not affected by the *ACTN3* genotype, but previous investigations have found that the XX genotype might be associated not only with a higher risk of non-contact muscle injuries but also with a longer recovery time [12]. 

### 4.3. Study Strengths

This study encompasses several strengths that indicate a step forward from previous investigations. First, this study has been carried out in a homogeneous sample of top-level football players competing in one of the most selective sports competitions. At present, humans are protected from the vagaries of nature, and the forces of natural selection are less strong in modern humans than those that our ancestors withstood. For this reason, *LaLiga* presents a perfect scenario to study natural selection in our society, as the great economic salary that football players obtain by playing in *LaLiga* shows that only the most talented players reach this level. The second strength is associated with the sample size, recruited with respect of the targeted population, as we recruited 315 players, who represent 65.6% of the total number of players in *LaLiga* 2021–2022 season (12 out of 20 teams agreed to participate in this investigation). This is particularly important as most of the investigations of genetics in sports are flawed because of the difficulty of obtaining DNA samples of true elite athletes. The last strength of this study is the assessment of phenotypes during real competition as athletic performance and injury were recorded during the official matches. Collectively, all of these strengths show that the outcomes of this study are of great practicality for physicians and football practitioners.

### 4.4. Study Limitations

First, although the accumulated match time over the season was similar across genotypes, there was no control of match exposure as the presence of each player in the team is an exclusive decision of the coach staff. So, this study did not completely fulfil the criteria of exchangeability required for strengthening outcomes of observational research in genetics [34]. The medical staff of the 12 clubs participating in this investigation had protocols to prevent and treat injuries based on FIFA guidelines [35]. However, these protocols were not identical as they were subjected to the preferences and experiences of each team’s staff. Therefore, reaching definitive conclusions about the association of the *ACTN3* genotype with injury incidence and time to return to play in professional football players may require replication in additional samples of top-level players. Additionally, future investigations should study the potential interaction of different genotypes in target polymorphisms, such as the I/D variation in the angiotensin-converting enzyme (*ACE*) gene [36], and the C-to-T polymorphism in the 3′-untranslated region of the collagen type V α1 chain (*COL5A1*) gene [37], as the current investigation only investigated one genetic variant in the sample of football players. Last, this study was carried out in a sample of only male professional football players, and the results of this study may not apply to female professional football players, as recently found for the Spanish *Liga F* [38]. Hence, the application of the results of this study to other populations of football players should be carried out with caution, as the conditions of *LaLiga* in terms of performance and competitiveness are unique.

## 5. Conclusions

In summary, the professional football players of *LaLiga* that possessed the *ACTN3* XX genotype had lower match running performance, especially in variables associated with high intensity running and sprinting, than their RR counterparts. Additionally, XX football players had a higher incidence of non-contact injury over the season, as they were more prone to injury during official matches and had a higher rate of muscle injury than RR players. If these causal links are confirmed by future investigations, it may suggest that α-actinin-3 deficiency due to the *ACTN3* XX genotype may constitute a factor with a negative impact on match running performance and injury incidence in male top-level football players.

## Figures and Tables

**Table 1:** Sociodemographic and match performance variables in 315 professional football players competing in *LaLiga* according to their *ACTN3* genotype.

Variable (Units)	RR	RX	XX	*p* Value
Sociodemographic variables				
Number (frequency, %)	116 (36.8)	156 (49.5)	43 (13.7)	-
Age (years)	26.8 ± 4.7	26.2 ± 4.7	26.8 ± 4.7	0.573
Height (cm)	181 ± 6	181 ± 6	182 ± 5	0.329
Body mass (kg)	76.0 ± 6.2	76.4 ± 7.3	77.7 ± 6.3	0.377
Body mass index (kg/m^2^)	23.3 ± 1.3	23.2 ± 1.4	23.3 ± 1.4	0.961
Ethnicity				
Caucasian (%)	97(33.7)	149 (51.7)	42 (14.6)	0.002
Afro-American (%)	19 (73.1) ↑	6 (23.1) ↓	1 (3.8)	
Asian (%)	0 (0.0)	1 (0.6)	0 (0.0)	
Match performance				
Total running distance (m/min)	113.8 ± 7.9	111.7 ± 9.4	109.0 ± 9.0 *	0.046
Distance at 2.0–6.9 km/h (m/min)	37.8 ± 2.8	37.6 ± 3.2	38.2 ± 2.6	0.711
Distance at 7.0–13.9 km/h (m/min)	42.1 ± 5.4	41.4 ± 6.1	39.9 ± 9.0	0.332
Distance at 14.0–20.9 km/h (m/min)	24.6 ± 4.8	23.3 ± 5.7	22.4 ± 6.3	0.139
Distance at 21.0–23.9 km/h (m/min)	4.0 ± 1.1	3.9 ± 1.2	3.5 ± 1.1 *	0.042
Distance at ≥24 km/h (m/min)	3.1 ± 1.4	3.1 ± 1.3	2.7 ± 1.4	0.400
Sprints (number/min)	0.23 ± 0.08	0.23 ± 0.09	0.20 ± 0.08 *	0.042
Mean sprint distance (m)	26.6 ± 1.2	26.4 ± 1.6	25.6 ± 1.2	0.653
Mean sprint duration (s)	5.1 ± 0.2	5.0 ± 0.3	4.9 ± 0.7 *	0.049
Peak running velocity (km/h)	30.1 ± 1.6	30.2 ± 1.4	29.8 ± 2.1	0.619
Match time (min)	1587 ± 852	1561 ± 876	1489 ± 902	0.704
Matches as starter (number)	17 ± 10	19 ± 10	20 ± 10	0.489
Matches as substitute (number)	7 ± 5	7 ± 5	5 ± 5	0.202
Training volume				
Total training time (min)	13,175 ± 1296	13,095 ± 1115	13,332 ± 1213	0.667

Match performance variables were obtained in a subsample of 259 football players after removing players with less than 90 min of match exposure during the whole season and goalkeepers. (↓↑) These arrows depict a statistically significant difference between the observed distribution and the expected distribution, according to the χ^2^ test and standardized residuals. The ↓ indicates that the observed distribution was lower than the expected distribution, and the ↑ indicates that the observed distribution was higher than the expected distribution, both at *p* < 0.050. (*) The asterisk depicts data with a statistically significant difference from players with the RR genotype at *p* < 0.050.

**Table 2:** Injury incidence in 315 professional football players competing in *LaLiga* depending on their *ACTN3* genotype.

Variable (Units)	RR	RX	XX	*p* Value
Injury incidence				
Total injury incidence (injury per 1000 h)	3.4 ± 3.9	4.9 ± 5.9	5.4 ± 4.2 *	0.035
Match injury incidence (injury per 1000 h)	18.9 ± 3.3	33.3 ± 7.6	43.8 ± 10.2 *	0.013
Training injury incidence (injury per 1000 h)	1.5 ± 2.7	1.6 ± 3.5	1.1 ± 3.0	0.718
Injury type				
Muscle injury (injury per 1000 h)	2.8 ± 3.8	4.2 ± 5.8	4.4 ± 4.2 *	0.021
Tendon injury (injury per 1000 h)	0.2 ± 1.2	0.3 ± 2.2	0.5 ± 2.1	0.565
Ligament injury (injury per 1000 h)	0.8 ± 1.8	0.4 ± 1.4	0.8 ± 2.3	0.713
Bone injury (injury per 1000 h)	0.1 ± 0.6	0.1 ± 0.6	0.4 ± 2.0 *	0.038
Cartilage injury (injury per 1000 h)	0.1 ± 0.8	0.1 ± 0.5	0.3 ± 1.9	0.517
Nerve injury (injury per 1000 h)	0.1 ± 6.9	0.1 ± 6.9	0.1 ± 5.9	0.997
Other type of injury (injury per 1000 h)	0.3 ± 1.3	0.4 ± 1.7	0.3 ± 1.9	0.808
Return to play				
Muscle injury (days)	16 ± 10	17 ± 12	20 ± 13	0.505
Tendon injury (days)	26 ± 32	26 ± 25	18 ± 23	0.549
Ligament injury (days)	54 ± 77	66 ± 67	54 ± 43	0.892
Bone injury (days)	11 ± 5	11 ± 5	29 ± 36	0.064
Cartilage injury (days)	18 ± 15	16 ± 19	13 ± 15	0.595
Nerve injury (days)	2	19	3	0.569
Other type of injury (days)	11 ± 3	11 ± 8	3 ± 3	0.565

Match injury incidence was obtained in a subsample of 259 football players after removing players with less than 90 min of match exposure during the whole season and goalkeepers. The * depicts a difference between players with XX genotype vs. players with RR genotype at *p* < 0.050, identified with one-way ANOVA.

## Data Availability

Restrictions apply to the availability of these data. Data were obtained from *LaLiga* and are available from the authors with the permission of *LaLiga*.
